# Kidney-Specific CAP1/Prss8-Deficient Mice Maintain ENaC-Mediated Sodium Balance through an Aldosterone Independent Pathway

**DOI:** 10.3390/ijms23126745

**Published:** 2022-06-16

**Authors:** Elodie Ehret, Yannick Jäger, Chloé Sergi, Anne-Marie Mérillat, Thibaud Peyrollaz, Deepika Anand, Qing Wang, Fréderique Ino, Marc Maillard, Stephan Kellenberger, Ivan Gautschi, Roman Szabo, Thomas H. Bugge, Lotte K. Vogel, Edith Hummler, Simona Frateschi

**Affiliations:** 1 Department of Biomedical Sciences, Faculty of Biology and Medicine, University of Lausanne, CH-1011 Lausanne, Switzerland; elodie.ehret@unil.ch (E.E.); yannick.jaeger@mpi-bn.mpg.de (Y.J.); chloesergi@gmail.com (C.S.); merillat10@gmail.com (A.-M.M.); thibaud.p@hotmail.com (T.P.); deepika.anand@unil.ch (D.A.); frederique.ino@unil.ch (F.I.); stephan.kellenberger@unil.ch (S.K.); ivan.gautschi@unil.ch (I.G.); simona.frateschi@epfl.ch (S.F.); 2 Department of Pharmacology, Max-Planck-Institute for Heart and Lung Research, D-61231 Bad Nauheim, Germany; 3 National Center of Competence in Research “Kidney.CH”, CH-1011 Lausanne, Switzerland; 4 Service of Nephrology, Department of Medicine, Lausanne University Hospital (CHUV), CH-1011 Lausanne, Switzerland; qing.wang@chuv.ch (Q.W.); marc.maillard@chuv.ch (M.M.); 5 National Institutes of Health, National Institute of Dental and Craniofacial Research, Bethesda, MD 20892, USA; rszabo@nidcr.nih.gov (R.S.); tbugge@dir.nidcr.nih.gov (T.H.B.); 6 Department of Cellular and Molecular Medicine, University of Copenhagen, DK-2200 Copenhagen, Denmark; vogel@sund.ku.dk; 7 Ecole Polytechnique Fédéral Lausanne (EPFL), CH-1011 Lausanne, Switzerland

**Keywords:** CAP1/Prss8, ENaC activation

## Abstract

The serine protease prostasin (CAP1/Prss8, channel-activating protease-1) is a confirmed in vitro and in vivo activator of the epithelial sodium channel ENaC. To test whether proteolytic activity or CAP1/Prss8 abundance itself are required for ENaC activation in the kidney, we studied animals either hetero- or homozygous mutant at serine 238 (S238A; *Prss8^cat/+^* and *Prss8^cat/cat^*), and renal tubule-specific CAP1/Prss8 knockout (Prss8^PaxLC1^) mice. When exposed to varying Na^+^-containing diets, no changes in Na^+^ and K^+^ handling and only minor changes in the expression of Na^+^ and K^+^ transporting protein were found in both models. Similarly, the α- or γENaC subunit cleavage pattern did not differ from control mice. On standard and low Na^+^ diet, *Prss8^cat/+^* and *Prss8^cat/cat^* mice exhibited standard plasma aldosterone levels and unchanged amiloride-sensitive rectal potential difference indicating adapted ENaC activity. Upon Na^+^ deprivation, mice lacking the renal CAP1/Prss8 expression (Prss8^PaxLC1^) exhibit significantly decreased plasma aldosterone and lower K^+^ levels but compensate by showing significantly higher plasma renin activity. Our data clearly demonstrated that the catalytic activity of CAP1/Prss8 is dispensable for proteolytic ENaC activation. CAP1/Prss8-deficiency uncoupled ENaC activation from its aldosterone dependence, but Na^+^ homeostasis is maintained through alternative pathways.

## 1. Introduction

Channel-activating proteases (CAPs) were shown to activate the epithelial sodium channel ENaC by increasing its open probability when co-expressed in vitro [1] and presented a novel autocrine regulation of ion channels [2]. CAP1/Prss8 (and its human orthologue prostasin) was the first protease shown to activate ENaC in vitro. This was followed by more members of the subfamily of membrane-bound serine proteases such as CAP2/Tmprss4 and CAP3/St14 (matriptase), and also intracellular or soluble serine proteases, like furin, elastase, kallikrein and plasmin as well as the metalloproteinase meprin or the lysosomal cysteine protease cathepsin-S [3,4]. In kallikrein-deficient mice, defective ENaC processing and function was documented in kidney cortex suggesting that the renal kallikrein-kinin system may play a role in Na^+^ balance and blood pressure regulation [5]. Any disruption in the system may result in human pathologies, including kidney injury and cardiovascular and inflammatory diseases [3,6]. ENaC mediates tubular Na^+^ absorption along the distal nephron thereby generating an electrochemical driving force for K^+^ secretion [3]. Deficiency of each of the three ENaC subunits in mice leads to deregulation of electrolyte homeostasis [7,8,9].

Several studies proposed prostasin a positive regulator of ENaC in the kidney, and thus as a candidate gene for hypertension in humans [10]. The expression of urinary CAP1/Prss8 is enhanced in rats infused with aldosterone and in patients with primary aldosteronism [11], possibly leading to increased ENaC-mediated Na^+^ reabsorption in the distal nephron. Adrenalectomy reduced urinary CAP1/Prss8 excretion to control levels [11]. Rats delivered with an adenovirus-mediated human prostasin showed a marked increase in blood pressure and electrolyte imbalance [12]. Olivieri and coworkers proposed urinary prostasin as candidate marker for ENaC activation in human and reported a positive correlation between urinary prostasin concentration, increased urinary excretion of the epithelial Na^+^ channel and primary aldosteronism [13,14]. Additionally, Li and coworkers reported an association of genetic variations of the prostasin gene with essential hypertension in the Xinjiang Kazakh ethnic population [15].

Synthesized as inactive zymogens, serine proteases act by cleaving proteins at their catalytic triad, composed of a serine, a histidine, and an aspartate. Proteolytic activation of ENaC may be achieved through intracellular cleavage of furin sites in the α and γENaC ectodomains followed by extracellular protease cleavage through candidate proteases like CAP1/Prss8, kallikrein or plasmin thereby leading to Na^+^ and water retention (see for review [4,16]. Indeed, Zachar and coworkers identified cleaved γENaC subunit in human kidney [17]. CAP1/Prss8 may thus extracellularly interact with the epithelial Na^+^ channel and facilitate cleavage of the γENaC-subunit to allow complete activation [16,18], although Mironova and Stockand found that the cleaved N-terminal domain of γENaC translocated to the nucleus and proposed that cleavage of ENaC may initiate a signaling cascade intracellularly [19]. The unspecific protease inhibitor aprotinin prevented proteolytic epithelial sodium channel activation and volume retention in a nephrotic mouse model and increased apical γENaC staining in kidney cortex was normalized by aprotinin treatment [20]. Essigke and coworkers proposed an essential scaffold function of prostasin acting to recruit another serine protease for proteolytic ENaC activation [21], but the consequences of a kidney-specific knockout of CAP1/Prss8 (prostasin) were not documented yet. 

To investigate cleavage and activation of ENaC by CAP1/Prss8 in the kidney, we used two CAP1/Prss8 mutant mouse models. First, mice harboring a S238A point mutation in the CAP1/Prss8 gene (Prss8^cat/cat^) that renders prostasin catalytically inactive and are healthy [22]. Constitutive *Prss8* knockout mice die during embryonic development [23], suggesting that prostasin exerts essential in vivo functions independent of its enzymatic activity. The conditional knockout of CAP1/Prss8 in lung and colon previously confirmed an in vivo regulatory role of prostasin in ENaC-mediated Na^+^ reabsorption [24,25,26], but this had not been yet addressed in kidney. We therefore included a second mouse model to study ENaC activation in a new inducible renal tubular specific CAP1/Prss8 knockout (Prss8^PaxLC1^).

## 2. Results

### 2.1. CAP1/Prss8-Deficiency Did Not Impair ENaC-Mediated Na^+^ Homeostasis

**Figure 1:**
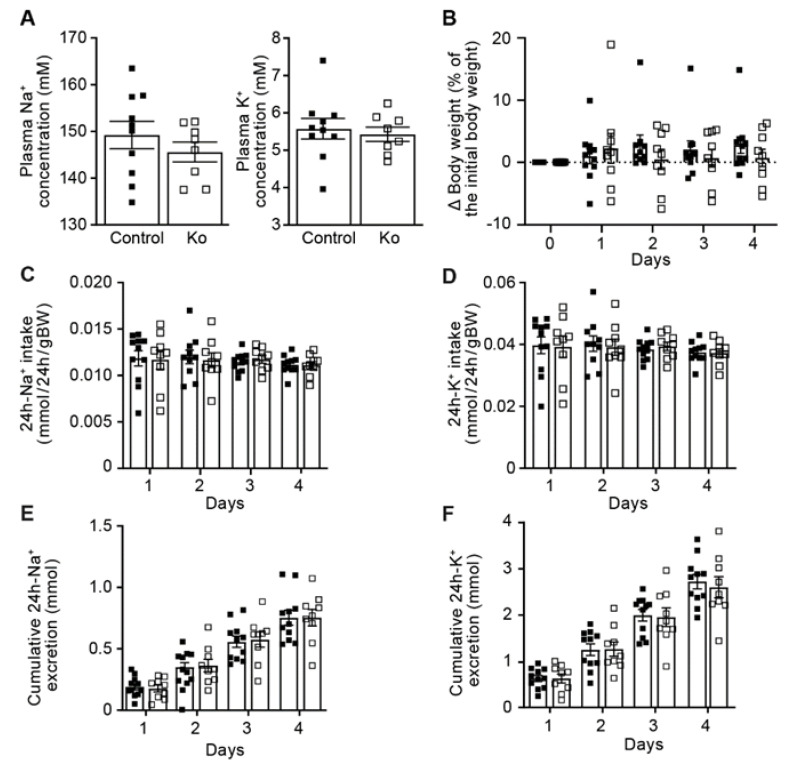
Prss8^PaxLC1^ (knockout) mice displayed normal Na^+^ and K^+^ handling under standard Na^+^ diet. (**A**) Plasma Na^+^ and K^+^ (mM) concentrations in control (left panel, black) and knockout (Ko, right panel, white square) mice. (**B**) Body weight changes (expressed as percent of initial body weight; control, n ≥ 10; Ko, n = 9), (**C**) 24h Na^+^ and (**D**) K^+^ intake and, (**E**) 24 h cumulative Na^+^ and (**F**) K^+^ (mmol/24 h/gBW) excretion; control, n = 11; Ko, n = 9). Results are presented as mean ± SEM (2 series of experiments, control: n = 10, knockout n = 8). (**A**) was analysed by an unpaired two-tailed *t* test, (**B**–**F**) by a multiple *t*-test. *p* values < 0.05 were considered statistically significant.

Macroscopically, the morphology of the adult kidney was not different between the control and knockout (Prss8^PaxLC1^) group (data not shown). To test whether renal proteolytic ENaC activation and Na^+^ handling was dependent on the presence of the serine protease CAP1/Prss8, we subjected inducible kidney specific CAP1/Prss8 knockout mice to standard diet. Adult kidney tubular-specific *Prss8* knockout (Prss8^PaxLC1^) mice appeared healthy (data not shown), showed normal plasma Na^+^ and K^+^ levels and were not affected in their body weight (Figure 1A,B). Their 24 h Na^+^ and K^+^ intake as well as their 24 h cumulative Na^+^ and K^+^ excretion did not differ from the control group (Figure 1C–F). Metabolic parameters like food and water intake, 24 h urine volume and feces as well as 24 h urinary Na^+^ and K^+^ excretions were not significantly changed in the knockout compared to the control group (data not shown). 

**Figure 2:**
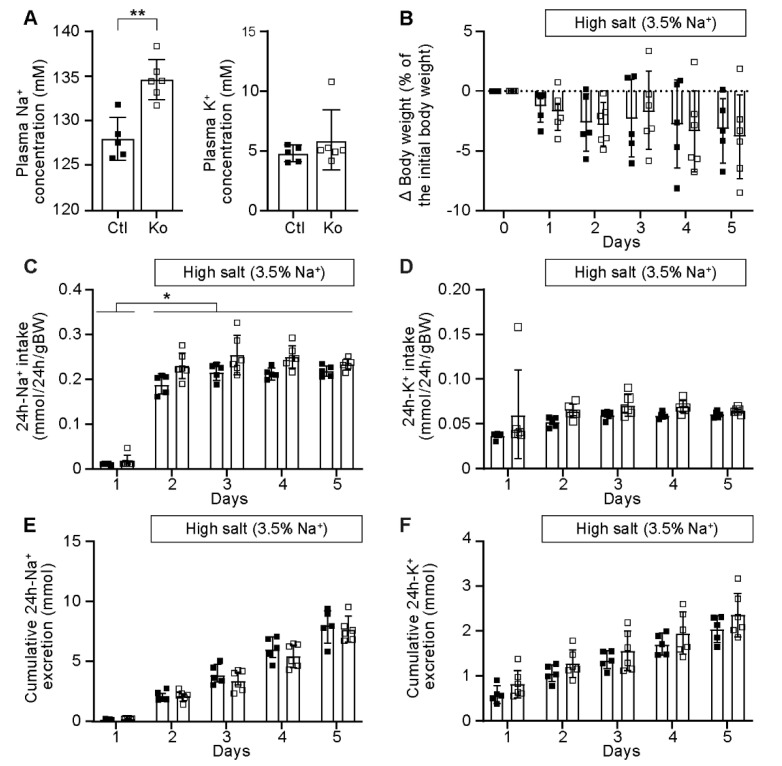
Prss8^PaxLC1^ (knockout) mice showed increased plasma Na^+^ concentration under high Na^+^ diet. (**A**) Plasma Na^+^ and K^+^ (mM) concentrations in control (n = 5, left panel, black) and knockout (Ko, n = 6, right panel, white square) mice. (**B**) Body weight changes (expressed as percent of initial body weight; control, n = 5; Ko, n = 6), (**C**) 24 h Na^+^ and (**D**) K^+^ intake and (**E**) 24 h cumulative Na^+^ and (**F**) K^+^ (mmol/24 h/gBW) excretion; control, n = 4–5; Ko, n = 5–6). Results are presented as mean ± SD. (**A**) was analyzed by unpaired two-tailed *t* test, (**B**–**F**) by a multiple *t*-test. *p* values < 0.05 were considered statistically significant. * *p* < 0.05, ** *p* < 0.01.

Following a four-day high Na^+^ diet, *Prss8* knockout mice exhibited increased plasma Na^+^ concentrations (Figure 2A) but did not show an impaired Na^+^ and K^+^ balance (Figure 2B–F) or altered metabolic parameters (data not shown).

**Figure 3:**
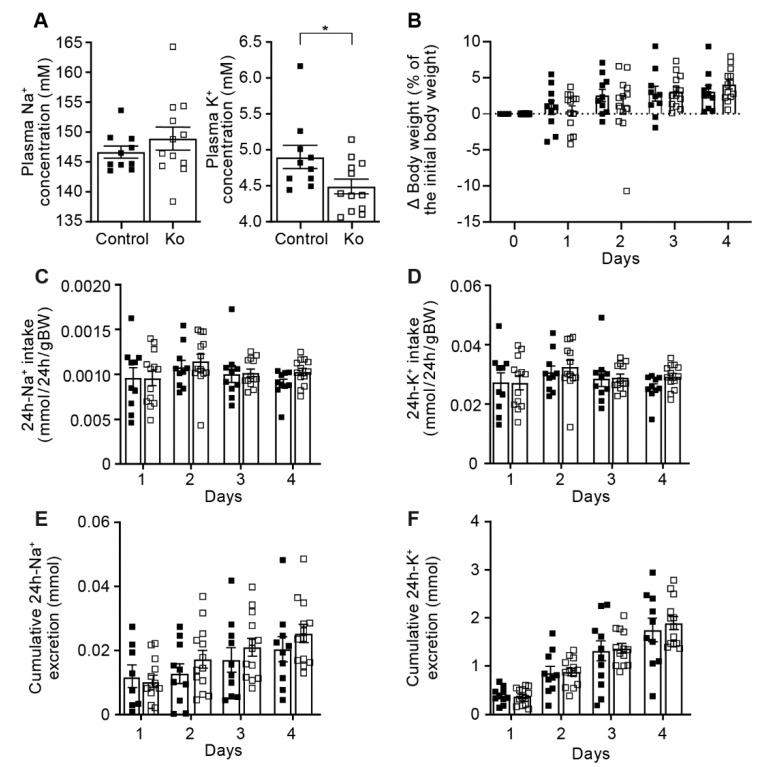
Prss8^PaxLC1^ (knockout) mice displayed normal Na^+^ but significantly altered K^+^ handling under low Na^+^ diet. (**A**) Plasma Na^+^ (left) and K^+^ concentrations (right) in control (black) and knockout (white square) mice. (**B**) Body weight changes (expressed as percent of initial body weight), (**C**) 24 h Na^+^ and (**D**) K^+^ intake and (**E**) 24 h cumulative Na^+^ and (**F**) K^+^ excretion (mmol/24 h/gBW; control, n = 9–10; Ko, n = 13). Results are presented as mean ± SEM (2 series of experiments, control n = 10, knockout n = 12). (**A**) was analyzed by an unpaired two-tailed *t* test, (**B**–**F**) by a multiple *t*-test. *p* values < 0.05 were considered statistically significant. * *p* < 0.05.

**Figure 4:**
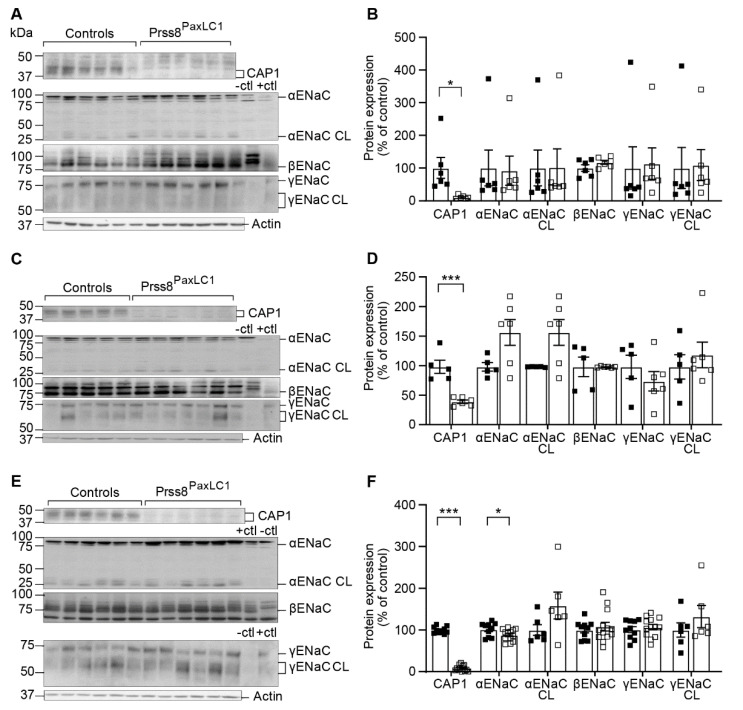
α- and γENaC subunits were still cleaved in Prss8^PaxLC1^ Ko mice under standard, high and low Na^+^ diet. Representative Western blot analyses of CAP1/Prss8, αENaC, αENaC CL (cleaved), βENaC, γENaC, γENaC CL (cleaved) of kidney lysates from control (black) and knockout mice (white square) under (**A**) standard, (**C**) high and (**E**) low Na^+^ diet.; β-actin was used as loading control. Kidney lysates of control and renal tubular-specific knockouts of αENaC [9], βENaC and γENaC [7,8] were used as negative (-ctl) and positive (+ctl) control. (**B**,**D**,**F**) Quantification of data. Values are mean ± SEM (2 series of experiments, control n = 10, knockout n = 13) and datasets were analyzed by an unpaired two-tailed *t* test. *p* values < 0.05 were considered statistically significant. * *p* < 0.05, *** *p* < 0.001.

We next challenged these mice with a low Na^+^ diet and, overall, Na^+^ and K^+^ handling was maintained as indicated by similar body weight, 24 h-Na^+^ and -K^+^-intake, cumulative 24 h-urinary Na^+^- and K^+^-excretion (Figure 3) and metabolic parameters like food and water intake, 24 h urine volume and feces as well as 24 h urinary Na^+^ and K^+^ excretions (data not shown). As potential compensatory mechanisms, we analyzed protein abundance of Na^+^ and K^+^-transporting systems, and found that, nearly independent of the diet, all ENaC subunits except αENaC that was reduced on low Na^+^ diet were similarly expressed in both groups (Figure 4A–F). We found minor changes in the protein abundance of NCC, pNCC, NHE3, NKCC2, ROMK or BK, namely a decrease in NKCC2 and ROMK on standard, a decrease in NCC and increase of NKCC2 in high Na^+^ diet and a decrease of BK on low Na^+^ diet (Figure S1). Overall, CAP1/Prss8 abundance seemed not to be required for ENaC-mediated Na^+^ and K^+^ homeostasis.

### 2.2. CAP1/Prss8 Was Not Required for Proteolytic ENaC Activation

We could not detect a significant reduction in the cleaved α(25kDa) and γ(75kDa) ENaC protein abundance independent of the Na^+^ diet (Figure 4). We therefore analysed whether proteolysis through CAP1/Prss8 is limiting for renal ENaC activity, and subjected mice mutated at serine 238 (S238A; *Prss8^cat^*^/+^, hetero- and *Prss8^cat/cat^*, homozygotes), as well as their littermate controls (*Prss8^+/+^*) to a standard (Figures S2 and S3) and low Na^+^ diet (Figures S4 and S5) followed by a detailed metabolic analysis. Under standard and low Na^+^ diet, heterozygous (*Prss8^cat/+^*) and homozygous mutant *Prss8^cat/cat^* mice showed no differences in Na^+^ and K^+^ handling compared to the control (*Prss8^+/+^*) mice as evidenced by normal plasma Na^+^ and K^+^ concentrations, similar body weight, 24 h-Na^+^ and K^+^ intake and cumulative 24 h-urinary Na^+^ and K^+^ excretion (Figures S2 and S4A–F). Upon both normal and low Na^+^ diet, colonic ENaC-mediated Na^+^ current in *Prss8^cat/cat^* mice did not differ from control (*Prss8^+/+^*) mice as evidenced by a similar amiloride-sensitive rectal potential (∆PDamil) difference (Figure S4H). Plasma aldosterone levels were not different between standard and low Na^+^ diet (Figure S2G; Figure S4G). Metabolic cage studies on standard and low Na^+^ diet did not reveal differences between *Prss8^+/+^*, *Prss8^cat/+^* or *Prss8^cat/cat^* mice for 24 h-food and water intake, urine volume, feces excretion, 24 h urinary Na^+^ and -K^+^ excretion (data not shown). 

We next studied kidney-tubular Na^+^ and K^+^-transporting proteins for potential compensatory mechanism. On all diet conditions, only minor changes in protein abundance were observed in heterozygous mutant *Prss8^cat/+^* mice with significant increased protein abundance for CAP1, pNCC and NKCC2, that however did not differ between *Prss8^+/+^* and *Prss8^cat/cat^* mutant mice on standard Na^+^ diet (Figure S3). On low Na^+^ diet (Figure S5), βENaC protein expression was significantly increased in *Prss8^cat/cat^* mice compared to *Prss8^+/+^* littermates, and ROMK protein abundance was increased in heterozygous (*Prss8^cat/+^*), but not different between *Prss8^cat/cat^* and *Prss8^+/+^* mice (Figure S5). No significant reduction in the cleaved α(25kDa) and γ (75kDa) ENaC protein abundance was observed (Figures S3 and S5). 

In summary, mice carrying a serine-mutant (S238A) within the catalytic triad of CAP1/Prss8, thereby rendering prostasin catalytically inactive, and mice lacking the renal tubular CAP1/Prss8 did not show impaired proteolysis of ENaC and overall maintained their Na^+^ and K^+^ balance. 

### 2.3. CAP1/Prss8-Deficiency Uncoupled Aldosterone Production from the Renin-Angiotensin-Aldosterone Pathway under Low Salt Diet

**Figure 5:**
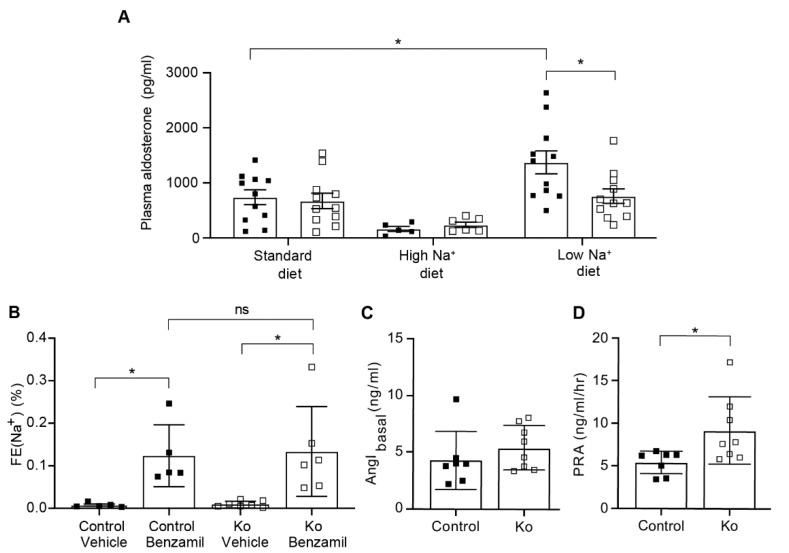
Prss8^PaxLC1^ Ko mice exhibited a decrease in plasma aldosterone concentration and an increase in plasma renin activity under low Na^+^ diet. (**A**) Plasma aldosterone levels in control (black square) and knockout mice (Ko, white square) under standard, high and low Na^+^ diet. (**B**) Natriuretic response expressed as fractional excretion of Na^+^ (in %) in control (black square) and knockout mice (Ko, white square) treated with vehicle or acute treatment of benzamil (0.2mg/kgBW). (**C**) Measurement of basal plasma angiotensin I concentration (determined at 4 °C) and (**D**), plasma renin activity (PRA) expressed as amount of angiotensin I following enzymatic cleavage of the renin substrate angiotensinogen (at 37 °C) in Prss8^PaxLC1^ control (black square) and knockout mice (Ko, white square). (**A**,**C**,**D**) were analyzed by an unpaired two-tailed *t* test. (**B**) was analyzed by a two-way ANOVA with a Sidak’s multiple comparisons test (column factor: genotype; row factor: treatment). (**A**) Results are represented as mean ± SEM (2 series of experiment, control n = 5–11, Ko n = 6–11). (**B**–**D**) Results are represented as mean ± SD. *p* values < 0.05 were considered statistically significant. * *p* < 0.05.

Since Prss8^PaxLC1^ knockout mice do not show any differences in proteolysis of ENaC and in urinary Na^+/^K^+^ levels, aldosterone levels were measured to monitor whether ENaC regulation is affected by the lack of CAP1/Prss8. Plasma aldosterone concentration was significantly lower in knockout mice under low Na^+^ diet compared to control mice (Figure 5A). To determine how decreased plasma aldosterone levels may account for the apparently increased ENaC activity upon Na^+^-deprivation, we next assessed the diuretic response in mice lacking renal CAP1/Prss8. Following Na^+^ deprivation, a single intraperitoneal injection of the specific ENaC blocker benzamil (0.2mg/kg) confirmed similarly increased ENaC activity measured indirectly as fractional excretion of plasma and urinary Na^+^ in control and knockout mice (Figure 5B). The natriuretic response to acute furosemide (NKCC2 inhibitor) and thiazide (NCC inhibitor) inhibition and the kaliuretic response to all three drugs was not different in vehicle- versus drug-treated control and knockout mice (data not shown). In contrast, while basal angiotensin I concentrations did not differ, plasma renin activity was significantly elevated in knockout mice (Figure 5) highly suggesting increased angiotensin II-stimulated ENaC activation (Figure 5C,D). 

**Figure 6:**
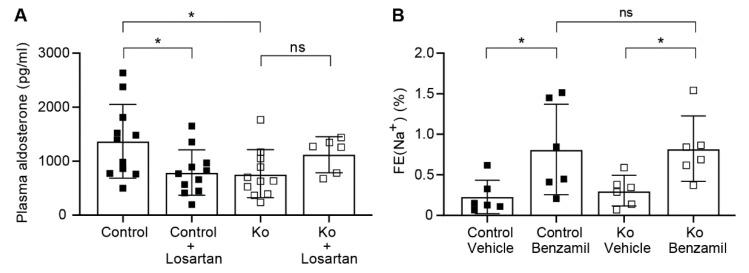
Under low Na^+^ diet, the production of aldosterone was uncoupled from the renin-angiotensin system in Prss8^PaxLC1^ Ko mice. (**A**) Plasma aldosterone levels in control (black square n = 11) and knockout mice (Ko, white square n = 6–11) under low Na^+^ diet and treated or not with losartan (10mg/kgBW) for 7 days. (**B**) Natriuretic response expressed as fractional excretion of Na^+^ (in %) in control (black square n = 6) and knockout mice (Ko, white square n = 6) treated with vehicle or acute treatment of benzamil (0.2mg/kgBW). All groups were treated during 7 days with losartan (10mg/kgBW). Results are presented as mean ± SD. (**A**) was analyzed by an unpaired two-tailed *t* test. (**B**) was analyzed with a two-way ANOVA with a Sidak’s multiple comparisons test (column factor: genotype; row factor: treatment). *p* values < 0.05 were considered statistically significant. * *p* < 0.05.

Application of losartan, an angiotensin II type 1 receptor blocker, did not change the plasma Na^+^ and plasma K^+^ concentrations between the control and knockout group on low Na^+^ diet, although there was a trend to lower plasma K^+^ levels in the losartan-treated knockout group (data not shown). Furthermore, the losartan treatment significantly reduced the plasma aldosterone concentration in control, but not in Prss8^PaxLC1^ knockout mice (Figure 6A). Following losartan treatment, a further benzamil-induced block of ENaC revealed equally increased fractional excretion of urinary Na^+^ indicative for a similar ENaC activity in both control and knockout mice (Figure 6B). 

To summarize, CAP1/Prss8 was not of key importance for proteolytic ENaC activation. However, following Na^+^ deprivation, mice lacking renal tubular CAP1/Prss8 showed an uncoupling of the aldosterone synthesis from the renin-angiotensin-aldosterone system, but maintained a Na^+^ and K^+^ balance. 

## 3. Discussion

### 3.1. CAP1/Prss8-Deficient Mice Preserved ENaC-Mediated Na^+^ Balance

To test the hypothesis that CAP1/Prss8 is required for the renal activation of ENaC, we studied the serine-mutant and the knockout of this serine protease in mouse models (Figure 1, Figure 2, Figure 3 and Figure 4). It was previously reported that co-expression of both wild-type and catalytically inactive CAP1/Prss8 activated ENaC in an aprotinin-sensitive manner in *Xenopus* oocytes [1,18]. Detailed metabolic and physiological analyses of the *Prss8^cat/cat^* knockin and the Prss8^PaxLC1^ knockout mice revealed that CAP1/Prss8 was not required for renal ENaC-mediated Na^+^ and K^+^ balance under standard, high and low Na^+^ diets (Figure 1, Figure 2 and Figure 3, Figures S2 and S4). Upon exposure to a Na^+^-deprived diet, mice harboring the S238A mutant (*Prss8^cat/cat^*) allele [22] showed no impairment in renal Na^+^ and K^+^ handling (Figures S2 and S4) and preserved proteolytic ENaC activation as evidenced by a similar α and γENaC cleavage pattern and a colonic diet-adapted ENaC activity in knockin mice comparable to control mice (Figures S3 and S5). Na^+^ balance was preserved independent of the catalytic activity of CAP1/Prss8 that is in agreement with recent findings in catalytically inactive CAP1/Prss8 mice under similar experimental conditions [21,27]. This raised the question whether CAP1/Prss8 might indirectly act on ENaC by recruiting a second yet unknown protease that finally facilitates γENaC cleavage [18,21]. Our data clearly showed that CAP1/Prss8 protein abundance was not required for the proposed scaffold function of ENaC activation (Figure 4) [21], nor is the expression and activity of common kidney Na^+^ and K^+^ transporting proteins severely affected (Figures S1, S3 and S5). 

Interestingly, CAP1/Prss8 deficiency in kidney did not induce a reduction in ENaC activity as seen in colonic and pulmonary CAP1/Prss8-deficient mice, although this reduction did not associate with a reduced cleavage profile of α and γENaC fragments [24,26] raising the question whether there is a direct correlation between total ENaC activity and the cleavage profile of the ENaC subunits. Pulmonary CAP1/Prss8-deficiency in mice impaired but not abolished the basal trans-epithelial alveolar Na^+^ (AFC) and water transport by about 50% which could be completely substituted by intra-alveolar treatment with neutrophil elastase [24]. In colon-specific CAP1/Prss8 knockout mice as well as in rats and mice with hypomorphic CAP1/Prss8 alleles, the amiloride-sensitive rectal potential difference was drastically reduced, but not completely abolished, and circadian cyclicity was blunted [25,26]. Alternatively, functional redundancy by other proteases might compensate for the deficiency of CAP1/Prss8. 

Indeed, several proteases, previously identified in vitro as activating the ENaC channel in *Xenopus* oocytes turned out to be dispensable for ENaC activation in vivo [3] although blocking serine protease activity by aprotinin, an unspecific serine protease inhibitor, prevented volume retention in a nephrotic mouse model, and inhibited proteolytic ENaC activation [20]. In the corresponding knockout animals, none of the tested channel-activating proteases revealed to be essential for renal ENaC activation [3,4]. The serine protease CAP2/Tmprss4 was instead involved in renal water handling upon dietary K^+^ depletion [28,29]. Mice lacking plasma kallikrein [30], or plasminogen/plasmin [31] or urokinase-plasminogen activator (uPA) were not protected from Na^+^ retention in experimental nephrotic syndrome [32], although Hinrichs and coworkers reported a urokinase-dependent intratubular plasminogen activation and γENaC cleavage in a podocin-deficient nephrotic mouse model [33].

Contrary to the CAP1/Prss8 deficiency in colon that becomes limiting for colonic ENaC activation, we observed no reduced ENaC activity following acute benzamil blockage on low Na^+^ diet conditions (Figure 5B) suggesting an additional compensatory mechanism of ENaC activation in the renal tubular specific CAP1/Prss8 knockout mice.

### 3.2. Upon Na^+^ Deprivation, CAP1/Prss8-Deficiency Resulted into an Aldosterone-Independent ENaC Activation

Following Na^+^ deprivation, plasma aldosterone and plasma K^+^ levels were low (Figure 5A), but plasma renin activity was significantly increased (Figure 5D) indicating that CAP1/Prss8 might be required for aldosterone dependent ENaC activation. Indeed, several previous studies proposed stimulation of ENaC by an acute stimulation of angiotensin II type 1 receptor (AT1R) [34,35]. Aldosterone-synthase knockout mice showed upregulation of the renin-angiotensin II system [36], and ENaC-mediated whole cell current in the DCT2/CNT was not further stimulated by a low Na^+^ diet which suggested that ENaC function is largely independent of aldosterone in this nephron segment [37]. In this context, it is interesting to note that αENaC heterozygous mutant mice developed an unusual mechanism to maintain blood pressure and Na^+^ balance by upregulating the number of angiotensin II type 1 receptor (AT1R) when studied on low Na^+^ diet [38]. Experimental animal models of angiotensin II-induced hypertension revealed an increased ENaC expression that might not be solely explained by increased secretion of angiotensin II-induced aldosterone. The effect of AT1R blockade on αENaC abundance was not prevented by spironolactone, a MR blocker, suggesting a direct implication of the receptor in αENaC gene expression [35]. Indeed, angiotensin II infusion in renal (MR) knockout mice had a large ENaC stimulatory effect while treatment with losartan inhibited ENaC in this latter segment suggesting a MR-independent ENaC activation [39]. Low plasma K^+^ might directly affect BK expression as observed in knockout mice on low Na^+^ diet (Figure S1E) and/or downregulate aldosterone which decrease BK expression. Unexpectedly, AT1R blockade by losartan treatment did not further reduce the plasma aldosterone concentration in knockout mice (Figure 6A) demonstrating that the production of plasma aldosterone is uncoupled from the angiotensin II levels in these mice, but the urinary fractional excretion of Na^+^ was similar in both control and knockout (Figure 6). In aldosterone-synthase knockout mice, diminished urinary K^+^ excretion, severe hyperkalemia, and food avoidance were observed [40], whereas our CAP1/Prss8 knockout mice showed normalized Na^+^ and K^+^ balance (data not shown) caused by low but still residual aldosterone concentration. Further compensation could be explained by an aldosterone-independent MR activation likely mediated by glucocorticoids in the presence of low 11β-HSD2 activity [41]. 

Interestingly, rats systemically overexpressing human prostasin showed the opposite phenotype to the knockout mice with a reduced plasma renin activity and increased plasma aldosterone levels [12]. On low Na^+^ diet, aldosterone-synthase (AS^−/−^) knockout mice showed more excreted Na^+^ after 1 day than after 1 week compared to the controls which could be compatible with two or more independent ENaC-activating pathways [42]. It was not reported, however, whether increased plasma renin activity was also implicated and/or decreased GFR was responsible for this phenomenon [42]. Several knockout models of the RAAS system, like e.g., mice deficient for SGK1 or aldosterone synthase showed only a minor salt-losing phenotype on low Na^+^ diet [43]. Potential stimulation of renin activity independent of aldosterone was often not considered. 

In this context, it is interesting to note that tissue kallikrein-deficient mice adapt normally to dietary sodium restriction despite absence of the 70 kDa γENaC cleaved form and reduced renal ENaC activity [5]. Enhanced expression of the renal NKCC2 transporter as seen in the CAP1/Prss8 knockout mice on high Na^+^ diet (Figure S1C,D) was not coupled with higher NKCC2 activity using furosemide blockage (data not shown). Higher NKCC2 activity has been linked to high salt sensitivity and hypertension although the mechanism is still largely unknown [44]. Due to the lack of specific protease inhibitors, it is currently difficult to determine the protease responsible for γENaC cleavage and/or activation. Interestingly, previous data of Narikiyo and co-workers claimed that aldosterone modified urinary excretion of CAP1/Prss8 [11] suggesting that CAP1/Prss8 could be the link between aldosterone and ENaC activation. It is interesting to note a significantly higher prevalence of the C allele of the CAP1/Prss8 (prostasin, rs12597511) gene in hypertensive pregnant women highly suggesting that this allele might present a risk for hypertension and cardiovascular diseases [45].

Overall, our data clearly showed that CAP1/Prss8 was not required for the direct proteolytic ENaC activation in kidney, and thus confirm data in lung- and colon-specific CAP1/Prss8 knockout mice [24,26]. In the absence of CAP1/Prss8, ENaC activity became limiting in lung and colon under challenging conditions, whereas renal ENaC activity was maintained most likely due to compensatory stimulating pathways. Na^+^ deprivation in CAP1/Prss8 knockout mice unveiled an uncoupling of the aldosterone production from the renin-angiotensin-aldosterone pathway, although ENaC-mediated sodium homeostasis is maintained through an aldosterone-independent mechanism. Future studies will show whether CAP1/Prss8 affects directly or indirectly the aldosterone production and/or secretion.

Our findings might be of importance for treatment of hypertensive patients with high renin levels, resistance to MR inhibition and ENaC-mediated sodium retention. Further investigations are needed to dissect this apparent complex aldosterone-dependent and -independent ENaC regulation in the control of Na^+^ and K^+^ balance that is highly dependent on diet conditions.

## 4. Materials and Methods

Heterozygous (*Prss8^cat/+^*) and homozygous mutant (*Prss8^cat/cat^*) prostasin (CAP1/Prss8) knockin mice carried a S238A substitution of CAP1/Prss8 on a C57BL/6J genetic background [22]. Age-matched female and male *Prss8^+/+^*, *Prss8^cat/+^* and *Prss8^catcat^* mice were obtained by interbreeding heterozygous mutant *Prss8*^cat/+^ mice to obtain control (Prss8^+/+^ and Prss8^cat/+^) and homozygous mutant Prss8^cat/cat^ mice. Inducible renal tubular-specific knockout mice (*Prss8^lox/lox^*; *Pax8-rTA^tg/0^*; *TRE-LC1^tg/0^*, herein termed Prss8^PaxLC1^*)* were obtained by intercrossing mice harboring the floxed CAP1/Prss8 allele (*Prss8^lox/lox^*) [46] and the transgenes *Pax8-rTA^tg/0^* or *TRE-LC1^tg/0^* [47] to obtain age-matched female and male control (*Prss8^lox/lox^*; *Pax8-rTA^tg/0^*; *TRE-LC1^0/0^*, *Prss8^lox/lox^*; *Pax8-rTA^0/0^*; *TRE-LC1^tg/0^* and *Prss8^lox/lox^*) and knockout (*Prss8^lox/lox^*; *Pax8-rTA^tg/0^*; *TRE-LC1^tg/0^*) mice. CAP1/Prss8 knockin (*Prss8^cat/+^* and *Prss8^cat/cat^*) mice were genotyped by PCR using ear biopsies (PRK1 forward (5′-GCAGCTCGAGGTACCACTCATCAG-3′) and PRK1 reverse primer (5′-AACTCACAATGCCTGCCAAGTACC-3′) [22]. The floxed *Prss8* control and knockout mice were genotyped as described [26]. PCR was run according to standard conditions (35 cycles, 1 min at 94 °C, 56 °C, and 72 °C). For breeding, mice were kept under a 14-h light and 10 h dark cycle and supplied *ad libitum* with food and water. At the age of 3 weeks, control and knockout (Prss8^PaxLC1^) mice were changed to a 12-h light/12-h dark cycle for 1 week before 10 days of induction by doxycycline (2 mg/mL doxycycline hydrochloride; Sigma, Deisenhofen, Germany) and 2% sucrose in the drinking water. Animal maintenance and experimental procedures were in agreement with the Swiss federal guidelines and were approved by the local committee for animal experimentation (Service de la Consommation et des Affaires Vétérinaires, Lausanne, Vaud, Switzerland) (#VD3333). The animals were housed in a temperature- and humidity-controlled room with an automatic 12-h light/dark cycle. If not indicated otherwise, 6–12-week-old age-matched male and female mice were fed on a standard (0.17% Na^+^), low (0.01% Na^+^) or high (3.5% Na^+^) diet (ssniff Spezialdiäten GmbH, Soest, Germany).

### 4.1. Western Blot Analyses

Kidneys and lungs were collected and snap frozen in liquid nitrogen and stored at −80 °C. Protein was extracted from frozen tissue as described [48] using a tissue lyzer (Qiagen). The following antibodies were used as previously described [49] diluted at a ratio of 1:500 (NCC, αENaC, NHE3), 1:1000 (βENaC, pNCC(T53), and 1:10,000 (NKCC2 [50]). Protein lysates from kidney tubular-specific α, β or γENaC knockout mice were used as a negative control [7,8,9]. Loading was controlled with β-actin. Antibodies for γENaC (StressMarq, Biosciences, Schweiz [39]) and NHE3/Slc9a3 [49] were diluted 1:500. or RomK/Kir1.1 (Alomone Labs, Jerusalem, Israel), and BK/Maxi-K, Kca1.1 (kindly provided by Olivier Staub, Lausanne, Switzerland) were diluted 1:10,000. Signals were revealed with ECL (Amersham ECL Western Blotting Detection Reagents, GE Healthcare, Chicago, IL, USA) and the band intensity was measured using the Image Studio Lite Software from LI-COR Biosciences or detected using film (Amersham hyperfilm ECL, GE healthcare). 

### 4.2. Metabolic Cage Studies

Following a standard (SD, 0.17% Na^+^), low Na^+^ (LS, 0.01% Na^+^, 10 consecutive days) or high Na^+^ (HS, 3.5% Na^+^, 4 consecutive days) diet in metabolic cages (Tecniplast, Buguggiate, Italy), physiological parameters as plasma Na^+^ and K^+^ concentration (mM), ∆body weight (% of the initial body weight at day 0 of the experiment), 24 h Na^+^ and K^+^ intake, urinary Na^+^ and K^+^ excretion (mmol/24 h/gBW), plasma aldosterone concentration (pg/mL), food and water intake (g/24 h/gBW), urine volume (ml/24 h/gBW), feces output (g/24 h/gBW), cumulative 24 h Na^+^ and K^+^ excretion (mmol) were determined. The amiloride-sensitive rectal potential difference (∆PDamil, mV) was measured as previously described with measurements taken at 4pm [25]. Following 2 days of adaptation, mice were maintained individually for further 4 days in metabolic cages. Blood and organ samples were collected at the end of experiment. Urinary and plasma Na^+^ and K^+^ concentrations were determined with the IL943 Flame Photometer (Instrumentation Laboratory, Cheshire, UK). Plasma aldosterone levels were measured using the Coat-A-Count RIA kit (Siemens Medical Solutions Diagnostics, Ballerup, Denmark) and the Aldo-Riact RIA (CISBio International, Saclay, France). Basal plasma angiotensin I concentration was measured using a radioimmunoassay (REN-CT2; CISBio International, Saclay, France). Quantitative plasma renin activity (PRA) is expressed as the amount of angiotensin I per ng produced per unit time (hour) and per mL of plasma following the enzymatic cleavage of the renin substrate, angiotensinogen, by the renin present in the test sample (after an incubation at 37 °C). The precision of these assays, based on intra- and interassay coefficients of variations are respectively 3.4% and 7.0% for PRA with a limit of quantification of 0.08 ng/mL/h and 2.8% and 8.7% for aldosterone with a limit of quantification of 18 pg/mL). Plasma aldosterone and angiotensin I and angiotensin II activity measurements were performed by the Service of Nephrology, (Lausanne University Hospital (CHUV), Lausanne, Switzerland [51].

### 4.3. Pharmacological Treatments 

To assess the natriuretic response, control and knockout (Prss8^PaxLC1^) mice were subjected to a low salt diet (0.01% Na^+^) during 10 days. Mice received intraperitonally (ip) a single dose of benzamil diluted in 0.5% DMSO + 0.9% saline (0.2μg/gBW, B2417, Sigma-Aldrich, St. Louis, MO, USA) [42], while controls received ip a single dose of vehicle (0.5% DMSO + 0.9% saline). Urine samples from both groups were collected during 6 h following the injection. Blood and organs were collected at the end of the experiment, and plasma and urinary Na^+^ excretion and creatinine were determined. Results are expressed as fractional excretion of Na^+^ as mean ± SEM. Following equation is used: FE_Na+_ = [(Urine_Na+_ × Plasma_creatinine_)/(Plasma_Na+_ × Urine_creatine_)] [52]. 

To block AT1R, control and knockout (Prss8^PaxLC1^) mice were subjected to a low Na^+^ diet (0.01%Na^+^) for 10 days. Following day 3 to day 10, mice received each day 10mg/kgBW of losartan (61188, Sigma-Aldrich) in drinking water. Blood was collected at the end of the experiment. Plasma aldosterone was measured by the Service of Nephrology, Lausanne University Hospital (CHUV), Lausanne, Switzerland [53].

### 4.4. Statistical Analyses

All statistical analyses were performed using GraphPad Prism and presented as scatter plot. To evaluate changes in the body weight of the mice, the water and food intake, the urine and feces excretion, the urinary electrolytes, the 24 h Na^+^ and K^+^ intake, the cumulative Na^+^ and K^+^, a multiple *t*-test was performed. To calculate the fractional excretion of Na^+^, a two-way ANOVA with a Sidak’s multiple comparison test (column factor: genotype; row factor: treatment) was made. For the *Prss8* control and knockout mice, quantification of Western blot analyses and plasma electrolytes were analyzed using an unpaired t-test and a one-way ANOVA with a Tukey test to compare *Prss8^+/+^*, *Prss8^cat/+^* and *Prss8^cat/cat^* mice. Results are represented as mean ± SEM or ± SD and a *p* value < 0.05 was considered statistically significant; * *p* < 0.05; ** *p* < 0.01, *** *p* < 0.001.

## Data Availability

The data that support the findings of this study are available from the corresponding author upon reasonable request.

## Supplementary Materials

The following supporting information can be downloaded at: https://www.mdpi.com/article/10.3390/ijms23126745/s1.
