# Supplementary material for: Kidney-Specific CAP1/Prss8-Deficient Mice Maintain ENaC-Mediated Sodium Balance through an Aldosterone Independent Pathway
Source: Int J Mol Sci. 2022 Jun 16;23(12):6745. doi: 10.3390/ijms23126745 (PMC9224322; doi:10.3390/ijms23126745)
Supplement: Supplementary file 1 [file ijms-23-06745-s001.zip › ijms-1754624-supplementary.pdf]

Appendix A

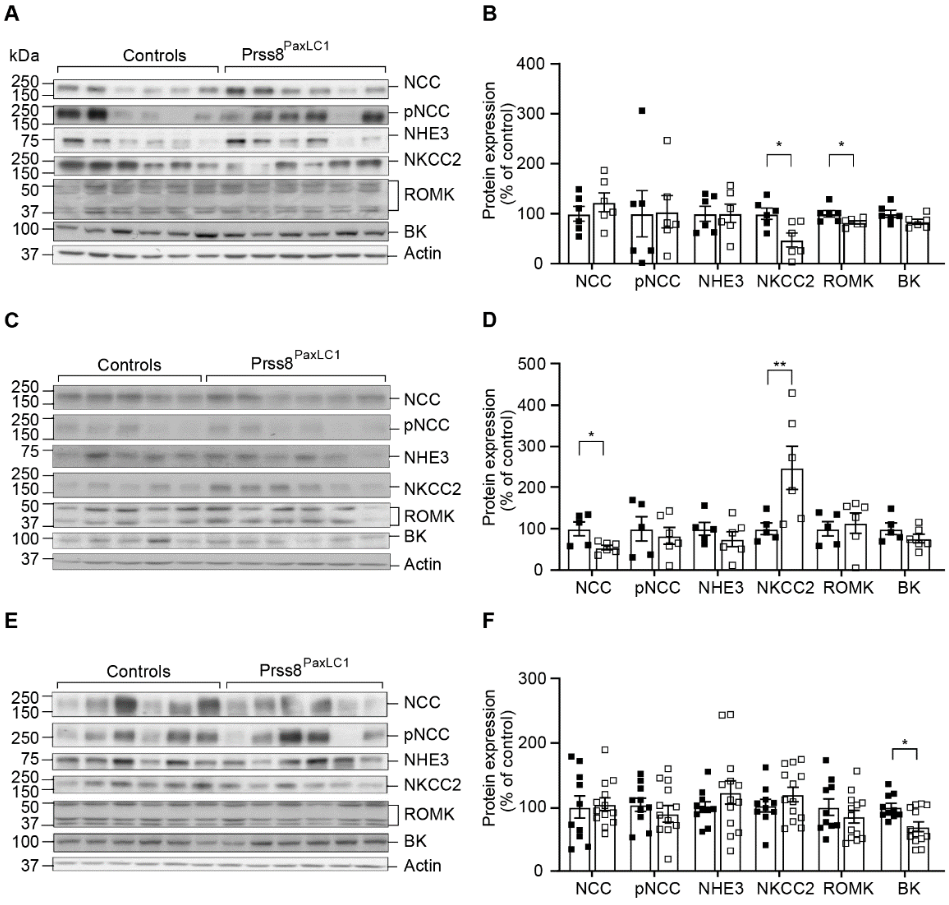

Figure S1. In Prss8^{PaxLC1} Ko mice, NKCC2 and ROMK protein expression levels were significantly reduced under standard, increased under high (only NKCC2) and not different on low Na^+ diet. Western blot analyses of NCC, pNCC (pT53-NCC), NHE3, NKCC2, ROMK and BK on kidney lysates from control and Prss8^{PaxLC1} Ko mice under **(A)** standard, **(C)** high and **(E)** low Na^+ diet.; β-actin was used as loading control. (**B,D,F**) Quantification of data. Values are mean ± SEM and datasets were analyzed by an unpaired two-tailed *t* test. *P* values <0.05 were considered statistically significant. **P* <0.05, ***P*<0.01.

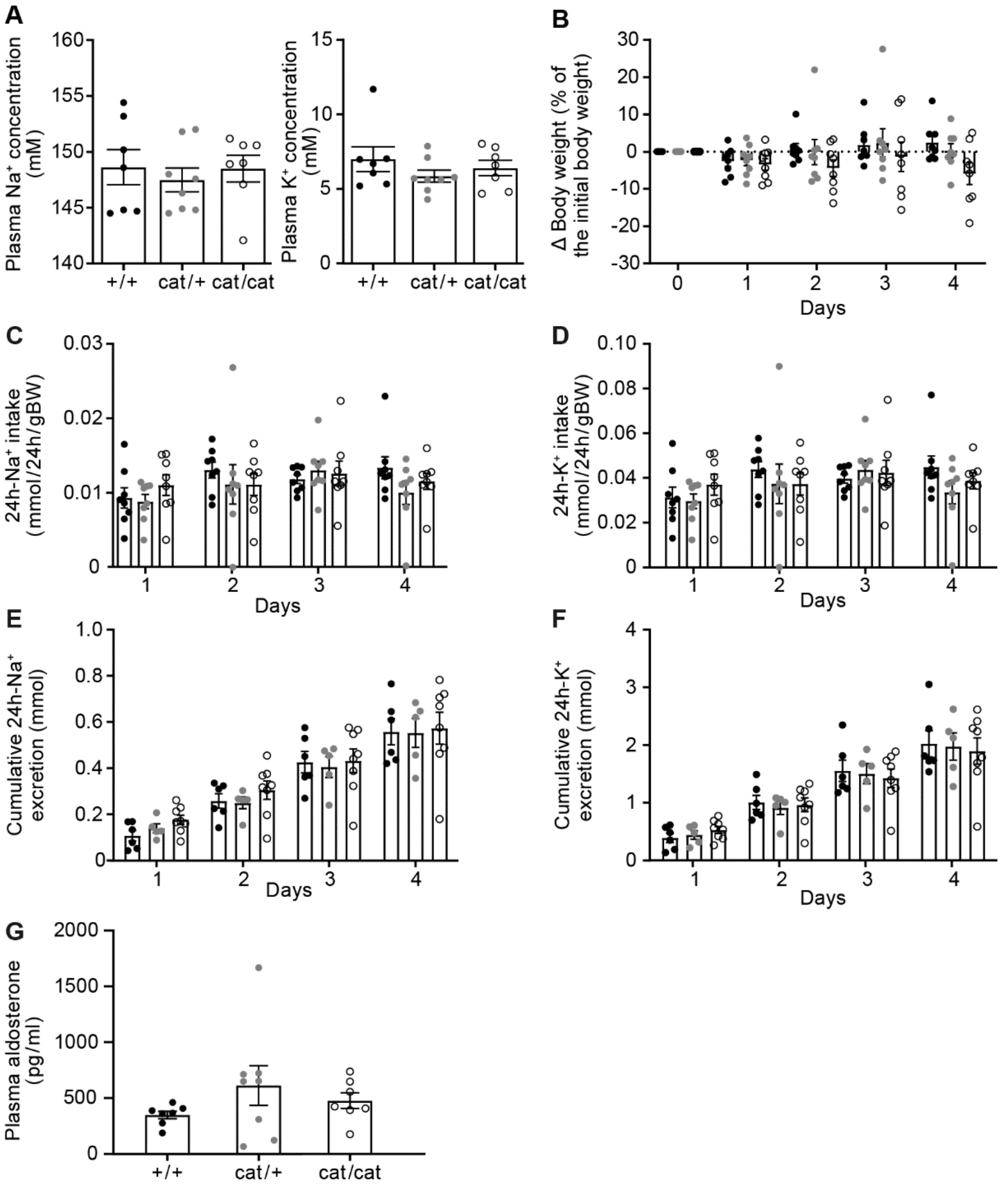

Figure S2. Under standard diet, heterozygous and homozygous mutant Prss8^{cat} mice displayed normal Na$^+$ and K$^+$ handling. (A) Plasma Na$^+$ and K$^+$ (mM) concentrations in control, heterozygous and homozygous Prss8^{cat} mice. **(B)** Body weight changes (expressed as percent of initial body weight, **(C)** 24h Na$^+$ and **(D)** K$^+$ (mmol/24h/gBW) intake and, **(E)** 24h cumulative Na$^+$ and **(F)** K$^+$ excretion (mmol/24h/gBW). **(G)** Plasma aldosterone levels. (+/+ n=7, *Prss8$^{cat/+}$* n= 8, *Prss8$^{cat/cat}$*, n=7). Results are presented as mean ± SEM. **A** and **G** were analyzed with a one-way ANOVA with a Tukey test. **B-F** were analyzed with a two-way ANOVA. *P* values <0.05 were considered statistically significant.

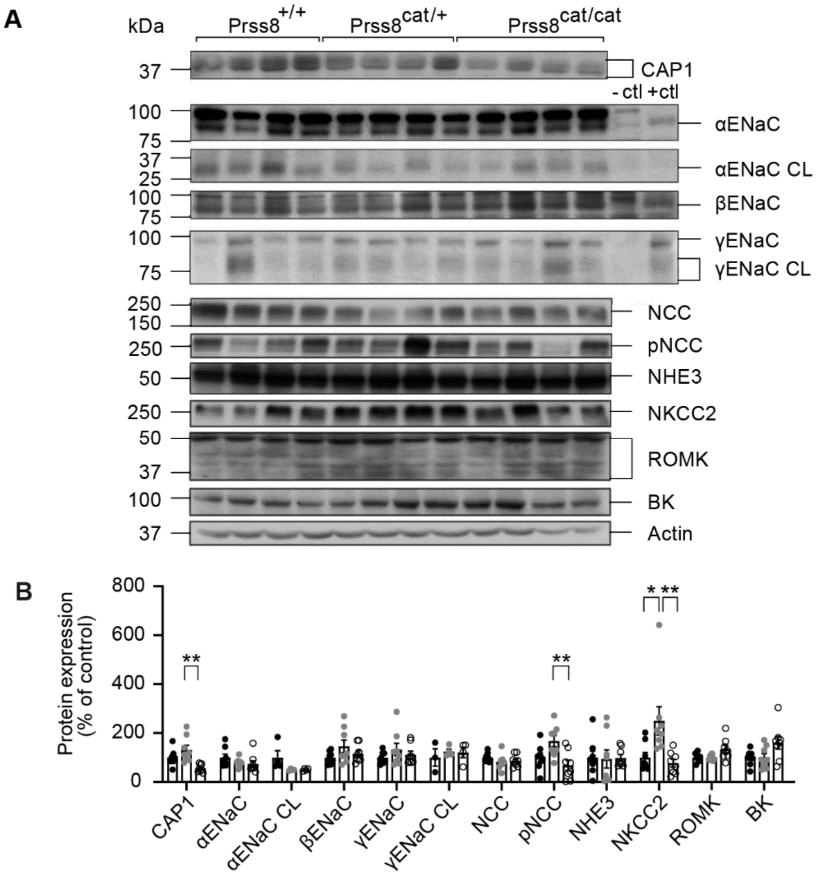

Figure S3. **Under standard diet, heterozygous mutant Prss8$^{cat/+}$ mice exhibited increased CAP1/Prss8, pNCC and NKCC2 protein expression levels.** (**A**) Representative Western blot analyses of CAP1/Prss8, αENaC, αENaC CL (cleaved), βENaC, γENaC, γENaC CL (cleaved), NCC, pNCC (pT53-NCC), NHE3, NKCC2, ROMK and BK on kidney lysates from wild-type (+/+) and heterozygous (*Prss8$^{cat/+}$*) and homozygous mutant *Prss8c$^{at/cat}$* (n=8 per genotype) littermates; β-actin was used as loading control. Kidney lysates from αENaC [9], βENaC and γENaC [7],[8] control and renal tubular-specific knockouts were used as negative (-ctl) and positive (+ctl) control. (**B**) Quantification of Western blots. Values are mean ± SEM and datasets were analyzed by a one-way ANOVA with a Tukey test. P values <0.05 were considered statistically significant. *P <0.05, **P <0.01.

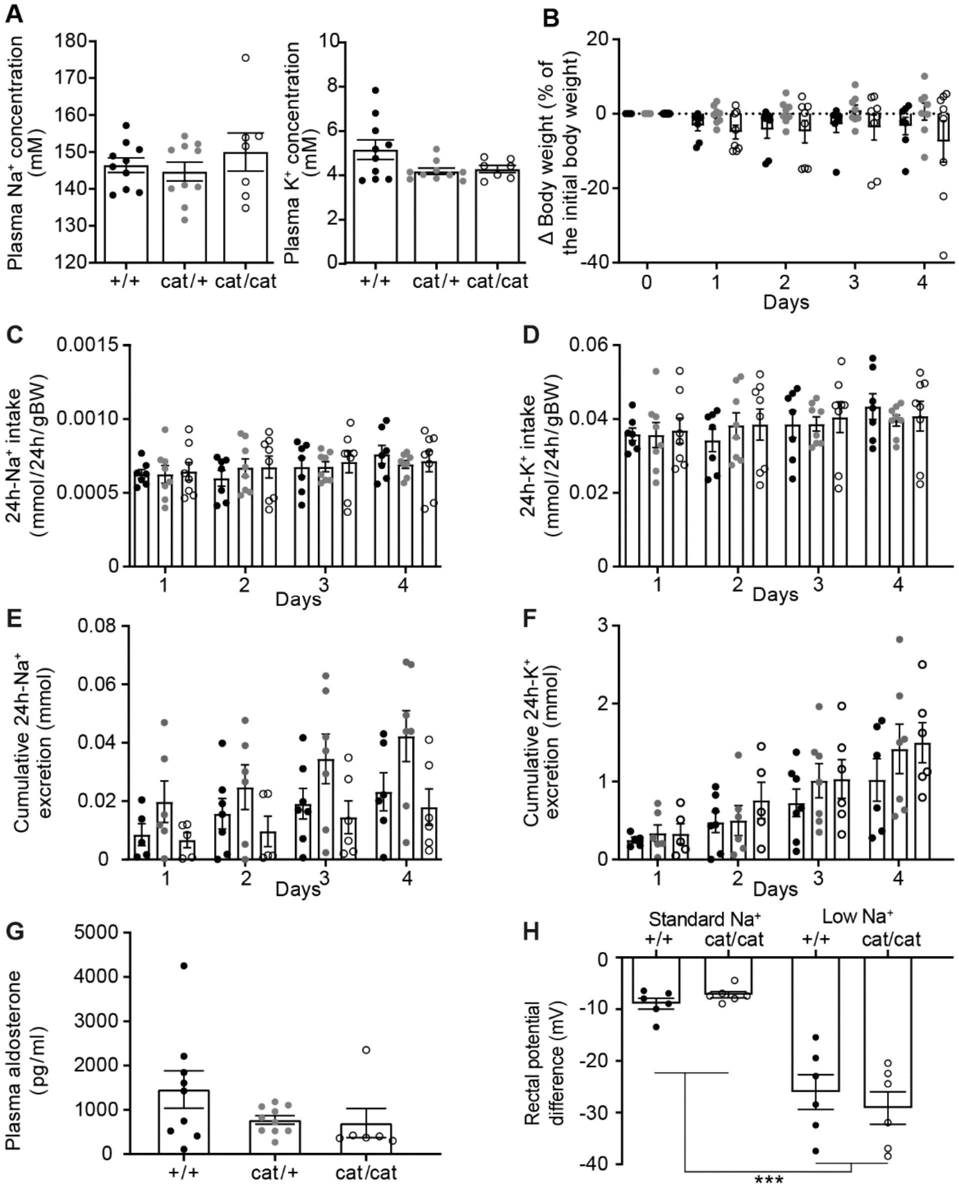

Figure S4. Under low Na+ diet, heterozygous (*Prss8*$^{cat/+}$) and homozygous mutant Prss8$^{cat/cat}$ mice displayed normal Na+ and K+ handling. (A) Plasma Na+ and K+ (mM) concentrations in control, heterozygous and homozygous Prss8^{cat} mice **(B)** Body weight changes (expressed as percent of initial body weight) **(C)** 24h Na+ and **(D)** K+ (mmol/24h/gBW) intake and **(E)** 24h cumulative Na+ and **(F)** K+ excretion (mmol/24h/gBW). **(G)** Plasma aldosterone levels. (+/+ n=5-10, *Prss8*$^{cat/+}$ n=6-10, *Prss8*$^{cat/cat}$, n=5-7). **(H)** Rectal ΔPD_{amil} (mV) measurements reflecting ENaC activity in wild-type (black) and homozygous mutant (white circle) *Prss8*$^{cat/cat}$ littermates in the distal colon following standard Na+ (left panel) and low Na+ diet (right panel). Results are presented as mean ± SEM, **A,G,H** were analyzed with a one-way ANOVA with a Tukey test**. B-F** were analyzed with a two-way ANOVA. P values <0.05 were considered statistically significant. ****P* <0.001

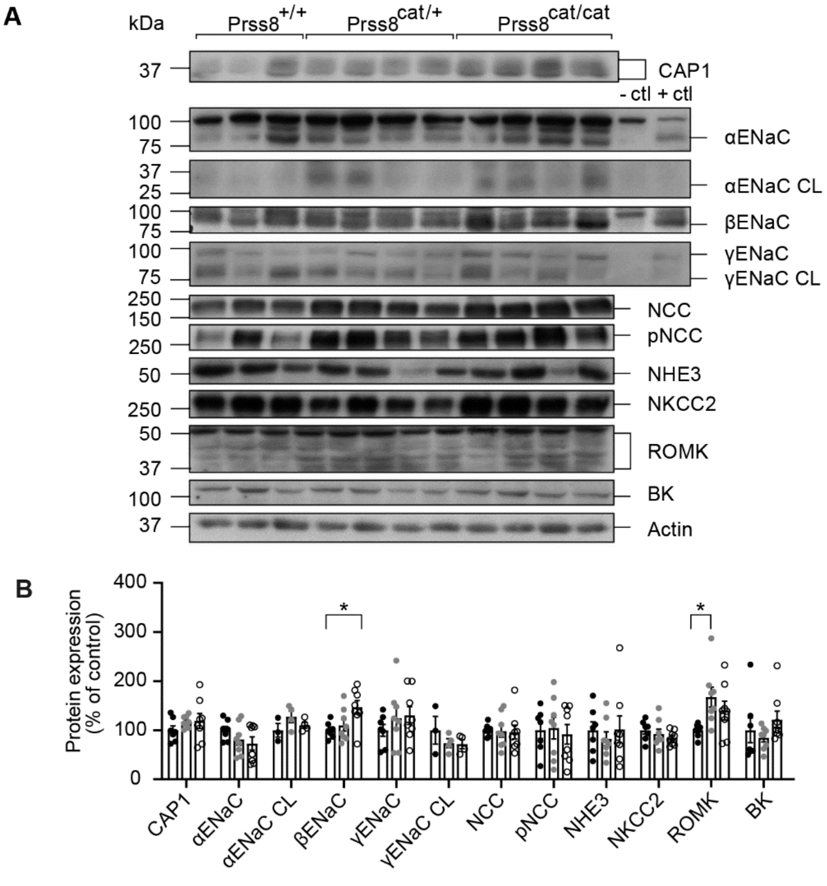

Figure S5. **Under low Na$^+$ diet, homozygous mutant Prss8$^{cat/cat}$ mice showed increased βENaC protein expression levels.** (**A**) Representative Western blot analyses of CAP1/Prss8, αENaC, αENaC CL (cleaved), βENaC, γENaC, γENaC CL (cleaved), NCC, pNCC (pT53-NCC), NHE3, NKCC2, ROMK and BK on kidney lysates from wild-type (+/+) and heterozygous (*Prss8$^{cat/+}$*) and homozygous mutant *Prss8$^{cat/cat}$* (n=8 per genotype) littermates; β-actin was used as loading control. Kidney lysates from αENaC [9], βENaC and γENaC [7],[8] control and renal tubular-specific knockouts were used as negative (-ctl) and positive (+ctl) control. (**B**) Quantification of Western blots. Values are mean ± SEM and datasets were analyzed by a one-way ANOVA with a Tukey test. *P* values <0.05 were considered statistically significant. *P<0.05
